# Novel expression of a functional trimeric fragment of human SP-A with efficacy in neutralisation of RSV

**DOI:** 10.1016/j.imbio.2016.10.015

**Published:** 2017-02

**Authors:** Alastair Watson, Nina Kronqvist, C. Mirella Spalluto, Mark Griffiths, Karl J. Staples, Tom Wilkinson, Uffe Holmskov, Grith L. Sorensen, Anna Rising, Jan Johansson, Jens Madsen, Howard Clark

**Affiliations:** a Clinical & Experimental Sciences Academic Unit, Southampton General Hospital, University of Southampton, Southampton, United Kingdom; b Division for Neurogeriatrics, Center for Alzheimer Research, Department of NVS, Karolinska Institutet, 141 57 Huddinge, Sweden; c Leukocyte Biology, Imperial College London, Royal Brompton Campus, London SW3 6NP, United Kingdom; d Southampton NIHR Respiratory Biomedical Research Unit, Southampton General Hospital, Southampton, United Kingdom; e Institute of Molecular Medicine, University of Southern Denmark, Odense, Denmark; f Institute for Life Sciences, University of Southampton, Southampton SO17 1BJ, United Kingdom; g Department of Anatomy, Physiology and Biochemistry, Swedish University of Agricultural Sciences, Box 7011, 750 07 Uppsala, Sweden

**Keywords:** Surfactant protein A, Recombinant trimeric fragment, Respiratory syncytial virus, NT domain, Innate immunity, Neutralisation, Collectin

## Abstract

Respiratory syncytial virus (RSV) is the leading cause of bronchiolitis and hospitalisation of infants in developed countries. Surfactant protein A (SP-A) is an important innate immune molecule, localized in pulmonary surfactant. SP-A binds to carbohydrates on the surface of pathogens in a calcium-dependent manner to enable neutralisation, agglutination and clearance of pathogens including RSV.

SP-A forms trimeric units and further oligomerises through interactions between its N-terminal domains. Whilst a recombinant trimeric fragment of the closely related molecule (surfactant protein D) has been shown to retain many of the native protein’s functions, the importance of the SP-A oligomeric structure in its interaction with RSV has not been determined.

The aim of this study was to produce a functional trimeric recombinant fragment of human (rfh)SP-A, which lacks the N-terminal domain (and the capacity to oligomerise) and test its ability to neutralise RSV in an *in vitro* model of human bronchial epithelial infection.

We used a novel expression tag derived from spider silk proteins (‘NT’) to produce rfhSP-A in *Escherichia coli*, which we found to be trimeric and to bind to mannan in a calcium-dependent manner. Trimeric rfhSP-A reduced infection levels of human bronchial epithelial (AALEB) cells by RSV by up to a mean (±SD) of 96.4 (±1.9) % at 5 μg/ml, which was significantly more effective than dimeric rfhSP-A (34.3 (±20.5) %) (p < 0.0001). Comparatively, native human SP-A reduced RSV infection by up to 38.5 (±28.4) %.

For the first time we report the development of a functional trimeric rfhSP-A molecule which is highly efficacious in neutralising RSV, despite lacking the N-terminal domain and capacity to oligomerise.

## 1 Introduction

RSV is the leading cause of acute childhood lower respiratory tract infection and a major cause of hospital admissions (Nair et al., 2010). Surfactant protein A (SP-A) is an important innate immune molecule expressed throughout the human respiratory tract and present in pulmonary surfactant. SP-A is a collectin, which binds to carbohydrates in a calcium-dependent manner and contains a collagenous region in common with other members of the collectin family such as both surfactant protein D (SP-D) and the serum collectin, mannan binding lectin (MBL). SP-A functions as an innate immune defence molecule, which binds to carbohydrates on the surface of an array of different pathogens, promoting their neutralisation, agglutination and clearance. SP-A has been shown to neutralise numerous different viruses such as RSV (LeVine et al., 1999) influenza A virus (Hartshorn et al., 1997) and HIV (Gaiha et al., 2008).

SP-A is also an important modulator of the inflammatory immune response, as previously reviewed (Wright, 2005). SP-A enhances uptake of apoptotic cells by macrophages and functions to modulate the production of pro-inflammatory mediators in a context dependent manner (Gardai et al., 2003). SP-A has been shown to enhance the killing of *Klebsiella pneumoniae* by macrophages (Kabha et al., 1997), modulate dendritic cell maturation (Brinker et al., 2003) and inhibit the proliferation and function of T cells (Borron et al., 1996, Borron et al., 2002). SP-A has also been shown to interact with various allergens (Malhotra et al., 1993) and prevent the binding of IgE from asthmatic children to house dust mite (Wang et al., 1996).

The potential importance of SP-A in RSV infection has been illustrated by the association of genetic polymorphisms within the SP-A genes with disease severity (Thomas et al., 2009, Ampuero et al., 2011, Lofgren et al., 2002, El Saleeby et al., 2010). However, the precise role of SP-A in neutralising RSV infection is not clear. *Sftpa1* knock out mice are shown to have increased titres of RSV in their lungs after infection as compared with wild type mice; treatment of these mice with exogenous SP-A enhanced RSV clearance (LeVine et al., 1999). Contrastingly, human SP-A has been reported to be exploited by RSV *in vitro* to provide a route of entry to enhance infection of Hep2 cells (Hickling et al., 2000). The importance of human SP-A in interacting with and neutralising RSV thus remains to be fully elucidated.

Human SP-A forms functional heterotrimeric units composed of *Sftpa1* and *Sftpa2* gene products (SP-A1 and SP-A2), the composition of which varies in different disease states (Tagaram et al., 2007). Each SP-A unit is composed of four domains: the functional trimeric lectin domain also known as the carbohydrate recognition domain (CRD); the alpha-helical neck domain responsible for trimerisation; a collagen-like domain and an N-terminal domain important for higher order oligomerisation. SP-A can oligomerise to form octadecameric structures, which resemble those of mannose-binding lectin (MBL). Oligomerisation increases the overall avidity of binding to polyvalent ligands and the capacity to agglutinate pathogens.

A functional recombinant fragment of human SP-D (rfhSP-D) has previously been produced. rfhSP-D contains only the CRD, neck and a short collagenous stalk but lacks the N-terminal domain and the majority of the collagen-like domain. This fragment has been well characterised, structurally and functionally (Clark et al., 2016). Using this molecule, it has been demonstrated that the full collagen domain and N-terminal domain of SP-D are not essential for many of the natural functions of SP-D. For example, rfhSP-D has been shown to be effective in neutralising a range of pathogens including RSV (Clark, 2010, Hickling et al., 1999). rfhSP-D has also been shown to be effective in both decreasing allergic inflammation and 1,3 β-glucan mediated neutrophilic inflammation and decreasing the degree of emphysematous change in SP-D^−/−^ mice (Fakih et al., 2015, Clark et al., 2005).

Technical problems have thus far impeded the production of an equivalent functional trimeric recombinant fragment of human SP-A (rfhSP-A). A recombinant fragment of rat SP-A has been previously produced (Head et al., 2003). However, the rat *Sftpa1* gene has only a 71% similarity to the human S*ftpa1* gene and functional differences between rat and human SP-A have been reported (Allen et al., 1999).

An equivalent fragment of SP-A would allow characterisation of the structure of the human SP-A CRD and the importance of the oligomeric structure for its native functions. Such a fragment would overcome previous problems associated with the full length SP-A with regards to self-aggregation of higher order oligomers mediated through the collagen-like domain (Haagsman et al., 1990). Moreover, it would overcome the requirement for expression in eukaryotic systems, which are expensive, and result in relatively low yields.

The N-terminal domain (NT) from spider silk and proteins (spidroins) is highly soluble on its own and allows high levels of soluble expression of spidroins (Kronqvist et al., 2014, Hedhammar et al., 2008, Rising and Johansson, 2015). This domain may have potential in allowing high levels of expression of other target proteins in heterologous systems. In this study, we investigated the use of NT of the major ampullate spidroin 1 from *Euprosthenops australis* as an expression partner to enable the production of a functional rfhSP-A molecule composed of SP-A1 (Rising et al., 2006). We used a human bronchial epithelial *in vitro* model to investigate the capacity of rfhSP-A to neutralise a clinically relevant strain of RSV as compared with native human (nh)SP-A.

## 2 Methods

### 2.1 Purification of nhSP-A

nhSP-A was purified from bronchoalveolar lavage fluid (BAL) from human patients with alveolar proteinosis using a butanol extraction method, as previously described (Wright et al., 1987). BAL was collected from patients at the Royal Brompton Hospital with informed consent and the necessary ethical permission (the Royal Brompton and Harefield Research Ethics Committee NRES 10/H0504/9).

### 2.2 Cloning

The *Sftpa1* (6A^2^) gene was cloned from human lung RNA. Ethical permission exists for the use of human lung tissue resected with informed consent from patients undergoing thoracic surgery at Southampton General Hospital (Southampton & SW Hants LREC 08/H0502/32). rfhSP-A was cloned into a pET 21a+ expression vector to include the CRD, neck and 8 x Gly Xaa Yaa repeats of the collagen stalk. The rfhSP-A gene was optimised for expression in *Escherichia coli* and subsequently sub-cloned into a pT7 vector containing the NT tag N-terminally of the rfhSP-A. A His_6_-tag was included N-terminally of NT to allow efficient purification and a thrombin cleavage site to allow removal of the NT tag after purification (Supplementary Fig. 1).

### 2.3 Expression of NT-rfhSP-A and isolation of inclusion bodies

BL21 (DE3) *E. coli* containing the plasmid encoding rfhSP-A or NT-rfhSP-A were grown in LB media containing appropriate antibiotics. Expression was induced by addition of IPTG (final concentration of 0.5 mM) and protein was expressed for 16 h at 30 °C. After lysis, inclusion bodies were isolated by centrifugation at 27,000 x *g*, 4 °C for 1 h and washed by suspension in 20 mM Tris 150, mM NaCl, pH 7.4 (TBS) containing 1% triton X-100 with subsequent centrifugation. This was repeated twice with the final wash being in TBS alone.

### 2.4 Purification of NT-rfhSP-A and subsequently rfhSP-A

NT-rfhSP-A was solubilised in 5 mM CaCl_2_ and 5% glycerol (v/v), 8 M urea, pH 7.4 (solubilisation buffer) at 4 °C, overnight with mixing. NT-rfhSP-A was refolded by dialysis at 4 °C for 2 h against solubilisation buffer but with decreasing concentrations of urea (4 M, 2 M, 1 M and 0 M). After removal of precipitate, NT-rfhSP-A was purified using an IMAC purification column and cleaved through incubation with 10 units of thrombin (GE Healthcare) per mg of protein for 6 h at room temperature. rfhSP-A was purified by reapplication to an IMAC column to remove His-tagged NT. NT-rfhSP-A and rfhSP-A were analysed by SDS-PAGE under reducing conditions with subsequent Coomassie staining or analysis by Western blotting using a monoclonal mouse IgG antibody raised against nhSP-A. rfhSP-A identity and purity was confirmed by mass spectrometry using previously described methods (Simon et al., 2016).

### 2.5 Gel permeation chromatography

The quaternary structure of rfhSP-A was characterised by gel permeation chromatography using an Äkta 900 system (Amersham BioSciences) with a 24 ml Superdex 200 HR 10/30 column, equilibrated in TBS with 5 mM EDTA, pH 7.4 (TBSE). The quaternary structure of rfhSP-A was estimated through comparison with elution positions of molecular weight standards kit (Sigma-Aldrich). Dimeric rfhSP-A was purified by gel permeation chromatography, as above but using a preparative 90 ml Superdex 200 column.

### 2.6 Purification of functional mannan binding rfhSP-A

Functional rfhSP-A was purified by affinity chromatography using a 15 ml mannan-coupled sepharose column. The affinity column was equilibrated in 20 mM Tris, 150 mM NaCl, 5 mM CaCl_2,_ pH 7.4 (TBSC). rfhSP-A was then injected onto the column using an Äkta 900 system. The column was washed in 20 mM Tris, 1 M NaCl, 5 mM CaCl_2,_ pH 7.4, after which it was re-equilibrated in TBSC. Functional rfhSP-A was eluted in TBSE.

### 2.7 Solid-phase mannan binding assay

Maxisorp plates were coated with 100 μl of mannan (50 μg/ml) in 0.1 M NaHCO_3_ pH 9.6 at 4 °C overnight. Plates were washed 4 times with TBS with 0.05% Tween (v/v) and blocked in TBS with 2% BSA (w/v) (block buffer) for 6 h at 37 °C. Protein was incubated at varying concentrations in either TBSC or TBSE at 4 °C overnight with subsequent washing in either TBSC or TBSE 4 times. Binding was detected using a polyclonal rabbit anti-nhSP-A IgG primary antibody and a goat anti-rabbit IgG HRP conjugated secondary antibody diluted in block buffer, with either 5 mM CaCl_2_ or 5 mM EDTA as appropriate. Wells were washed in either TBSC or TBSE 4 times, as above. SP-A binding was detected by addition of 3,3′,5,5′-tetramethylbenzidine (TMB) reagent mix with subsequent inhibition of reaction after 15 mins with 0.5 M H_2_SO_4_. Absorbance was measured at λ = 450 nm.

### 2.8 Infection of bronchial epithelial cells with RSV

Human bronchial epithelial cells (AALEB), immortalised through specific transfection with the simian virus 40 early region and the telomerase catalytic subunit hTERT, were used in infection assays and have previously been described (Lundberg et al., 2002). AALEB cells were grown in Bronchial Epithelial Growth Medium (BEBM plus SingleQuots of Growth Supplements) (Lonza). AALEB cells were grown to 80% confluency in 24 well plates coated with collagen. AALEB cells were serum starved for 24 h in BEBM supplemented with ITS 1X (insulin, transferrin, selenium, Thermo Fisher Scientific) and 0.02% BSA (Sigma-Aldrich) and infected with a clinically relevant RSV-A (Memphis 37) strain, originally isolated by DeVincenzo et al. (DeVincenzo et al., 2010). Cells were infected with either a low (multiplicity of infection (MOI) of 0.08) or high (MOI of 0.4) dose of RSV diluted in DMEM (4 mM l-glutamine). RSV was preincubated with varying concentrations of nhSP-A, rfhSP-A, dimeric rfhSP-A or BSA diluted in DMEM (4 mM l-glutamine). Cells were infected for 2 h, after which they were washed and left for 24 h in BEGM media without serum but with recommended supplements.

### 2.9 Quantifying RSV infection by RT-qPCR

RNA was harvested from cells infected at an MOI of 0.08 using peqGOLD TriFast (Peqlab, Germany), according to manufacturer’s instructions. Reverse transcription was performed using a High-Capacity cDNA Reverse Transcription Kit (Life Technologies) with random primers according to manufacturer’s instructions. RSV N gene expression was analysed using TaqMan Universal PCR Master Mix (No AmpErase UNG reagent) with an Applied Biosystems 7900HT Fast Real-Time PCR System machine (all from Life Technologies). Gene expression was normalised against expression of Hypoxanthine Phosphoribosyltransferase 1 (HPRT) using the 2^−ΔCt^ method. Average relative percentage infection was then calculated by normalisation as a percentage against the RSV untreated control.

### 2.10 Flow cytometry

Cells infected with an MOI of 0.4 were detached from wells using trypsin-EDTA (Sigma-Aldrich), washed in PBS and fixed in Cytofix/Cytoperm (BD Biosciences) at 4 °C for 20 min. Infected cells were identified using a mouse anti-RSV-F protein IgG primary antibody (Ambsio: C01626M) and a goat anti-mouse IgG antibody conjugated with Alexa-Fluor 488 secondary (Invitrogen: A11001) diluted in Perm/Wash (BD Biosciences). Cells were analysed using a FACSAria cell sorter (BD Biosciences). Cells were regarded as infected if above the fluorescence threshold, which was set to approximately 1% of the uninfected control. Average relative percentage infection was calculated as above.

### 2.11 Statistical analysis

An unpaired two-tailed Student’s *t*-test with equal variance was used to calculate differences of RSV infection by treatment with protein. To calculate significant differences between treatments, a two-way ANOVA with multiple comparisons was used corrected using the Bonferroni method. Results were regarded as statistically significant at p < 0.05.

## 3 Results

### 3.1 The NT tag allows high levels of rfhSP-A expression and purification

**Fig. 1:**
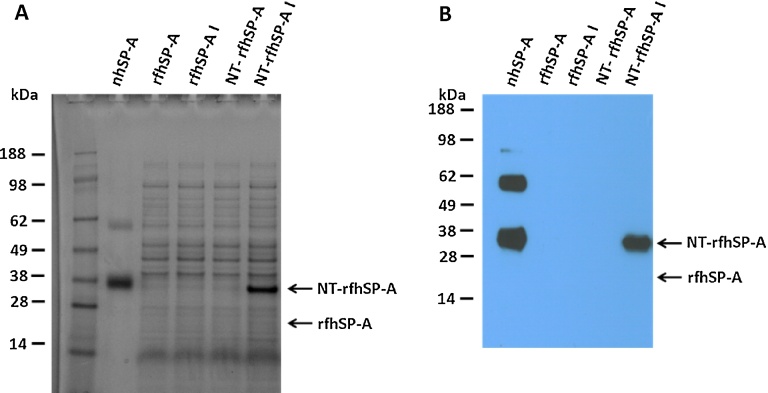
rfhSP-A expression with and without the NT solubility tag. rfhSP-A and NT-rfhSP-A expression was induced using 0.5 mM IPTG overnight at 30 °C. Expression was analysed by SDS-PAGE under reducing conditions with subsequent (A) Coomassie staining or (B) Western blotting analysis using an antibody raised against SP-A. Indicated are the bacterial samples before induction (rfhSP-A and NT-rfhSP-A) and post induction (rfhSP-A I and NT-rfhSP-A I). nhSP-A was also included as a positive control for comparison with Western blotting.

**Fig. 2:**
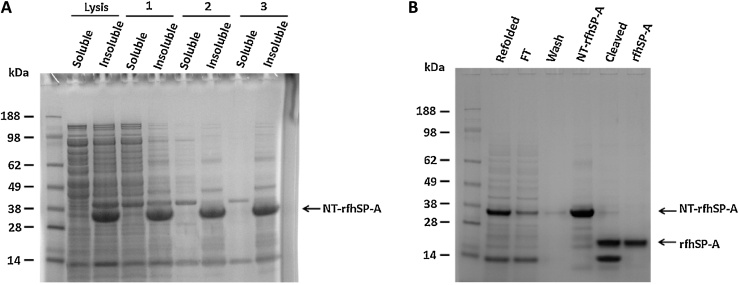
NT-rfhSP-A is expressed as an insoluble protein but allows isolation of pure rfhSP-A. Purification samples were analysed by SDS-PAGE with subsequent Coomassie staining. (A) NT-rfhSP-A was expressed in inclusion bodies as indicated by the analysis of soluble and insoluble fraction of cell lysate after increasing numbers of washes (1, 2 and 3) of inclusion bodies. (B) Analysis of rfhSP-A purification samples after solubilisation and subsequent refolding (refolded), flow through of the nickel column (FT), washing of nickel column (Wash), purification of target fusion protein (NT-rfhSP-A), cleavage of target fusion protein (Cleaved) and purification of rfhSP-A from cleaved NT-rfhSP-A (rfhSP-A).

To produce a recombinant trimeric fragment of human SP-A, *E. coli* bacteria were initially inoculated with the expression plasmid containing the 8x Gly Xaa Yaa triplets, neck and CRD of human *Sftpa1*. However, upon induction, the rfhSP-A protein was not expressed at detectable levels (Fig. 1). Different expression temperatures, times and IPTG concentrations did not improve expression, which could only be detected by overexposure of a western blot (Supplementary Fig. 2). Implementation of the novel expression tag NT, however, overcame this problem and allowed high levels of expression of an NT and rfhSP-A fusion protein (NT-rfhSP-A) (Fig. 1). NT-rfhSP-A was expressed in inclusion bodies as indicated by the analysis of soluble and insoluble fractions. Inclusion bodies containing NT-rfhSP-A were washed and NT-rfhSP-A was solubilised using 8 M urea, with subsequent refolding (Fig. 2A). The refolded NT-rfhSP-A was purified effectively using nickel affinity chromatography with subsequent removal of the NT tag. This led to the generation of pure rfhSP-A with no NT tag contamination (Fig. 2B).

### 3.2 The rfhSP-A sequence is sufficient to form carbohydrate binding trimeric units

**Fig. 3:**
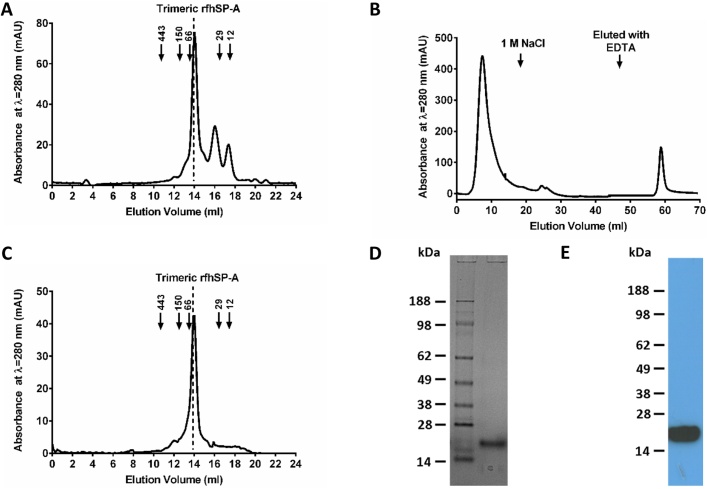
Purification of functional trimeric rfhSP-A. (A) rfhSP-A was analysed by gel permeation chromatography. Elution of protein was detected by measuring optical absorbance at λ = 280 nm. Indicated are the elution volumes of molecular weight standards: apoferritin 443 kDa; alcohol dehydrogenase 150 kDa; bovine serum albumin 66 kDa, carbonic anhydrase 29 kDa and cytochrome C 12 kDa. rfhSP-A is mainly trimeric and the trimer eluted from the column at 13.8 ml (expected molecular weight of trimer, 57 kDa). (B) Functional rfhSP-A was purified by mannan affinity chromatography. rfhSP-A was eluted from the mannan affinity column after washing in 1 M NaCl with 5 mM CaCl_2_ using TBS with 5 mM EDTA. (C) Functional rfhSP-A purified by mannan affinity chromatography was analysed by gel permeation chromatography as above. Purified functional rfhSP-A was assessed by SDS-PAGE under reducing conditions with (D) Coomassie staining and (E) Western blotting analysis using an antibody raised against nhSP-A.

Purified rfhSP-A was analysed using gel permeation chromatography (Fig. 3A). 72% of the purified rfhSP-A was trimeric, with 15% being dimeric and 9% being monomeric protein, highlighting that the CRD, neck and 8x Gly Xaa Yaa sequence of human SP-A is sufficient to form trimeric units. A proportion of rfhSP-A was functional and bound to a mannan coupled affinity column in a calcium-dependent manner; use of carbohydrate affinity chromatography thus allowed functional carbohydrate binding protein to be purified (Fig. 3B). The rfhSP-A purified by mannan affinity chromatography was trimeric (Fig. 3C) and of high purity as assessed by SDS-PAGE (Fig. 3D), Western blotting (Fig. 3E) and mass spectrometry (data not shown). The trimeric structure was stable upon further analysis by gel permeation chromatography after freeze/thawing. Dimeric rfhSP-A was likewise purified by gel permeation chromatography for comparison, and did not bind to a mannan coupled affinity column (Supplementary Fig. 3). Comparatively, purified nhSP-A was of higher order oligomeric structure with an apparent weight of >669 kDa as compared to molecular weight standards (data not shown).

**Fig. 4:**
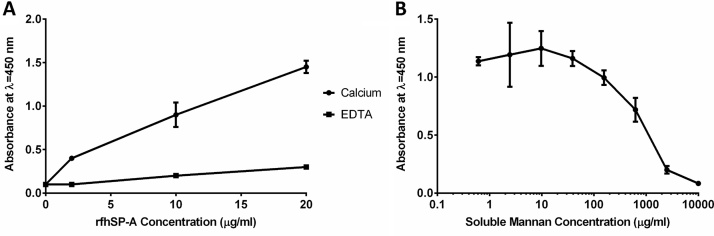
Confirmation of trimeric rfhSP-A being functional in binding to mannan. (A) Various concentrations of rfhSP-A were applied to mannan coated plates in the presence of calcium or EDTA. Binding was detected using an antibody raised against nhSP-A. (B) Specificity of binding to mannan was confirmed by addition of 5 μg/ml of trimeric rfhSP-A in the presence of calcium with increasing amounts of soluble mannan. Displayed are mean ± SD of 3 experiments.

The capacity of carbohydrate affinity purified trimeric rfhSP-A to bind to mannan was confirmed using a mannan solid-phase binding assay (Fig. 4A). Trimeric rfhSP-A bound to mannan in a calcium-dependent manner. This binding was specific to the mannan coated onto the plates and was inhibited by the presence of soluble mannan (Fig. 4B); nhSP-A also bound mannan coated plates in a calcium dependent manner (data not shown).

### 3.3 rfhSP-A is highly efficacious at neutralising RSV

**Fig. 5:**
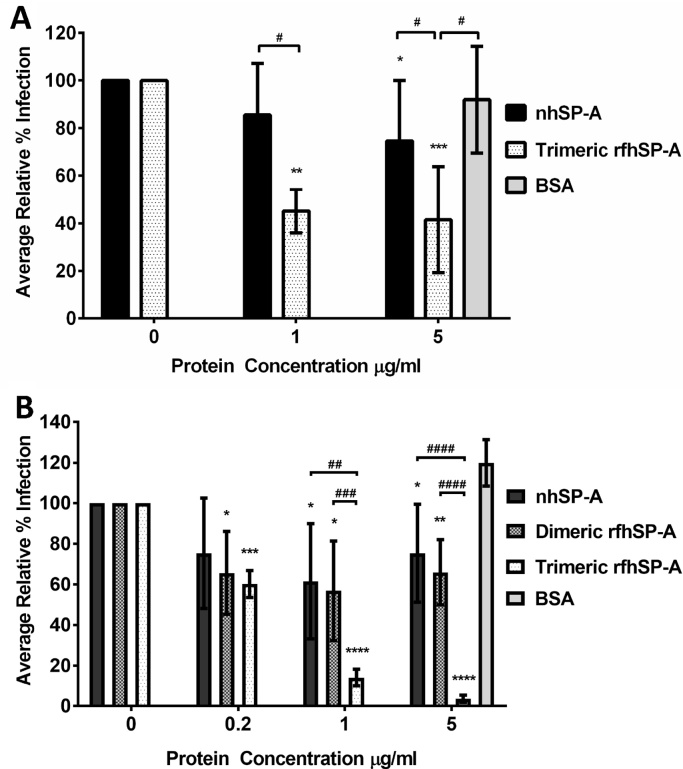
Trimeric rfhSP-A reduces RSV infection of human bronchial epithelial cells. (A) RSV N gene expression was quantified using RT-qPCR in human bronchial epithelial (AALEB) cells infected with a low dose of RSV (MOI of 0.08). Prior to infection, RSV was incubated for 1 h at 37 °C either alone or with 1 μg/ml or 5 μg/ml of nhSP-A or trimeric rfhSP-A or 5 μg/ml of bovine serum albumin. (B) Infection levels of AALEB cells infected with a higher dose of RSV (MOI of 0.4) were quantified by flow cytometry using an antibody raised against RSV F protein. Prior to infection, RSV was incubated for 1 h at 37 °C either alone or with 0.2 μg/ml, 1 μg/ml or 5 μg/ml of nhSP-A, trimeric rfhSP-A, dimeric rfhSP-A or BSA. Shown is the mean (±SD) of at least 3 experiments undertaken in duplicate. Indicated are significant differences between untreated and treated virus (calculated using unpaired two tailed Student’s *t*-test with equal variance) (* p < 0.05, ** p < 0.01, *** p < 0.001, **** p < 0.0001) and significant differences between treatments (calculated using two-way ANOVA with multiple comparisons corrected using the Bonferroni method) (# p < 0.05, ## p < 0.01, ### p < 0.001, #### p < 0.0001).

The capacity of trimeric rfhSP-A to neutralise RSV and prevent infection of differentiated human bronchial epithelial (AALEB) cells was compared with nhSP-A at a low dose of RSV (MOI of 0.08). RSV RNA was quantified by RT-qPCR at 24 h after infection (Fig. 5A). Pre-incubation of RSV with nhSP-A reduced RSV infection by a mean (±SD) of 14.5 (±21.6) % (not significant (n.s)) at 1 μg/ml and significantly by 30.0 (±22.8) % (p < 0.05) at 5 μg/ml. Comparatively, pre-treatment with trimeric rfhSP-A significantly reduced infection in a dose-dependent manner by 54.9 (±9.0) % (p < 0.01) and 63.7 (±22.2) % (p < 0.001) at 1 μg/ml and 5 μg/ml, respectively. Pre-treatment of RSV with 5 μg/ml of BSA did not reduce infection levels.

To confirm the functionality of trimeric rfhSP-A and its capacity to neutralise RSV, AALEB cells were infected using a higher dose of RSV (MOI of 0.4) and virus presence was detected using flow cytometry (Fig. 5B). Bronchial epithelial cells were gated by size and width (Supplementary Fig. 4A) and were infected in a dose dependent manner with increasing titres of virus. An MOI of 0.4 resulted in ∼30–35% of cells being detected as infected as determined by flow cytometry; only background levels of infection were detected upon infection with UV treated RSV (Supplementary Fig. 4). Pre-incubation with nhSP-A reduced RSV infection by a mean (±SD) of 24.7 (±27.2) % at 0.2 μg/ml (n.s) and significantly by 38.5 (±28.4) % and 24.7 (±29.6) % at 1 μg/ml and 5 μg/ml, respectively (p < 0.05) (Fig. 5B). Trimeric rfhSP-A significantly reduced RSV infection in a dose-dependent manner by 39.8 (±6.8), 85.9 (±4.2) and 96.4 (±1.9) % at 0.2 μg/ml 1 μg/ml and 5 μg/ml, respectively (p < 0.001, p < 0.001 and p < 0.0001, respectively). Importantly, at 5 μg/ml trimeric rfhSP-A reduced relative infection levels to only 3.7 (±2.2) % (p < 0.0001), thus reaching base line levels in uninfected controls (2.5 (±0.2) % (p = 0.2)). Dimeric rfhSP-A also reduced RSV infection to some degree by 34.3 (±20.5) %, 43.2 (±24.5) % and 34 (±16.1) % at 0.2 μg/ml, 1 μg/ml and 5 μg/ml, respectively (p < 0.05, p < 0.05 and p < 0.01, respectively). However, trimeric rfhSP-A was significantly more effective at reducing RSV infection at 1 μg/ml and 5 μg/ml than dimeric rfhSP-A (p < 0.001 and p < 0.0001, respectively). Contrasting to pretreatment of RSV with nhSP-A, trimeric and dimeric rfhSP-A, pre-incubation with 5 μg/ml of BSA did not reduce infection levels.

## 4 Discussion

SP-A has been shown to interact with and neutralise RSV *in vivo*. However, the importance of the human SP-A oligomeric structure in its interaction with RSV has not previously been determined. We have introduced a novel expression system to overcome previous technical issues to generate for the first time a functional trimeric rfhSP-A molecule and demonstrated its efficacy in neutralising RSV.

### 4.1 Production of a functional trimeric rfhSP-A molecule using a novel expression tag (NT)

The generation of recombinant versions of SP-A has been important in delineating the anti-pathogenic and immunomodulatory functions of SP-A (Wright, 2005). Recombinant SP-A may have therapeutic potential, particularly as an adjunct treatment to current lipid surfactants alongside recombinant SP-D. These recombinant collectins could replace the deficient immunomodulatory host proteins SP-A and SP-D in the premature neonatal lung and prevent the development of neonatal chronic lung disease with associated respiratory and neurological complications (Clark, 2010). Recombinant SP-A and SP-D may also have potential as novel adjunctive synthetic anti-inflammatory and anti-infective agents in other disease settings including severe asthma and COPD (Clark, 2010, Mackay et al., 2016).

Previous studies have produced full-length recombinant human SP-A molecules (Head et al., 2003, Garcia-Verdugo et al., 2003). However, problems with full length SP-A have been found with regards to self-aggregation mediated through the collagen-like domain and higher order oligomers (Haagsman et al., 1990). Full length SP-A requires expression in eukaryotic systems, which is expensive, and results in relatively low yields. A trimeric rfhSP-A molecule which lacks the majority of the collagen domain and is expressible in bacteria would overcome these issues.

One study reported an attempt to produce a truncated fragment of human SP-A without the collagen stalk but did not demonstrate the production of a functional trimeric fragment (Sotiriadis et al., 2015). The inclusion of the 8x Gly Xaa Yaa collagen stalk in the related rfhSP-D molecule, is thought to stabilise the trimeric structure, and has previously been shown to be essential for its function *in vivo* (Knudsen et al., 2009). In addition, removal of the entire SP-A collagen domain through collagenase digestion has previously been shown to result in either purely monomeric subunits (Murata et al., 1993) or a mixture of trimers and monomers, dependent on the buffer salt concentration (Haagsman et al., 1989). A short collagen stalk may therefore be required for a functional trimeric rfhSP-A molecule.

Previous attempts to express a trimeric rfhSP-A molecule including the 8x Gly Xaa Yaa collagen stalk have not been successful. This could be for various reasons, including potential difficulty of translating the N-terminal part of the truncated protein or the presence of numerous prolines, as found in rfhSP-A: this has previously been reported to have a negative impact on elongation of protein translation in *E. coli* (Hersch et al., 2014). Through implementing a novel *E. coli* expression strategy using a new heterologous expression tag, NT, we have overcome the issues of expressing trimeric rfhSP-A with the collagen stalk and for the first time demonstrated the production of a functional trimeric rfhSP-A molecule.

In nature, NT allows expression of large amounts of soluble spidroins and has allowed expression of a very aggregation-prone amyloidogenic protein (Hedhammar et al., 2008, Dolfe et al., 2016). Using this expression tag, we have expressed trimeric rfhSP-A at high levels in a bacterial expression system. Importantly, comparative to full length recombinant SP-A molecules, trimeric rfhSP-A lacks the majority of the collagen domain and the N-terminal domain and thus has a lower propensity to self-aggregate, and has an increased solubility. The rfhSP-D of the closely related molecule SP-D is a well characterised molecule and has provided a wealth of information about the structure/function relationship of SP-D and mode of calcium-dependent ligand binding (Clark et al., 2016, Clark et al., 2005, Clark et al., 2003, Clark, 2010, Strong et al., 2002, Lin et al., 2010, Knudsen et al., 2007, Roona et al., 2012). Thus, this functional trimeric rfhSP-A may prove a useful reagent for research and has increased potential for development as a therapeutic as compared with full-length recombinant SP-A.

### 4.2 Trimeric rfhSP-A lacking the N-terminal domain is highly effective at neutralising RSV

In this present study, we have demonstrated the capacity of both nhSP-A and a functional trimeric rfhSP-A molecule to neutralise a clinically relevant strain of RSV in an *in vitro* human bronchial epithelial cell model. This suggests that similarly to SP-D, the N-terminal domain and entire collagen domain is not required for neutralisation of RSV (Hickling et al., 1999).

Strikingly, trimeric rfhSP-A reduced RSV infection to levels near to the uninfected control. Thus the N-terminal domain and majority of the collagen domain is not essential for the capacity of SP-A to neutralise RSV (Barr et al., 2000). nhSP-A has previously been shown to neutralise RSV and reduce infection levels by 13.3% and 53.3% at a concentration of 10 μg/ml and 20 μg/ml, respectively (Ghildyal et al., 1999). In this present study, lower concentrations of nhSP-A were used but the capacity for neutralisation was not dissimilar with infection levels being reduced by up to 38.5 (±28.4) % at 1 μg/ml. nhSP-A significantly reduced RSV infection compared to both preincubation without protein or preincubation with a BSA control.

Trimeric rfhSP-A appeared to neutralise RSV more effectively than oligomeric nhSP-A. This increased efficacy could in part be due to the lower molecular weight of rfhSP-A and thus increased number of functional CRDs per microgram of protein. With the molecular weight of a nhSP-A subunit being 26–38 kDa comparative to the 19 kDa molecular weight of a rfhSP-A unit, there were up to 2 fold more CRDs for each treatment with trimeric rfhSP-A compared with nhSP-A. However, this does not fully account for the increased efficacy of treatment with trimeric rfhSP-A particularly upon infection with the higher dose of RSV (Fig. 5B). In this study, nhSP-A was purified from patients with alveolar proteinosis using butanol extraction. Although this method has been widely used in the literature for purifying nhSP-A, functionality could be impacted by the specific patient from which the SP-A was purified, the absence of the lipid surfactant or the extraction method itself (Wright et al., 1987, Tino and Wright, 1999, Tino and Wright, 1996, McKenzie, 2015, Whitwell et al., 2016). However, nhSP-A used in this study was confirmed to be oligomeric and functional in binding to mannan.

Upon increasing the dose of nhSP-A from 1 to 5 μg/ml, the levels of RSV neutralisation were not significantly increased. This also suggests that the increased efficacy of trimeric rfhSP-A as compared with nhSP-A is not solely a consequence of the number of functional CRDs in the assay. The apparent increased efficacy of nhSP-A at neutralising the higher dose of RSV at a concentration of 1 μg/ml as compared with 5 μg/ml is difficult to explain but could simply be due to 1 μg/ml being a sufficient dose to reduce RSV infection by the maximum amount; any slight difference between 1 and 5 μg/ml could be due to experimental variability. Alternatively there could be dual mechanisms in play. SP-A has previously been reported to be exploited by RSV *in vitro* and to provide a route of entry to enhance infection of Hep2 cells (Hickling et al., 2000). Thus, it is tempting to hypothesise that nhSP-A could work in a dual manner to both neutralise RSV to some degree but also interact with putative receptors to provide a route of entry into the cell, the balance of these two mechanisms could be highly dependent on the concentration of nhSP-A. The N-terminal domain of SP-A is thought to interact with numerous receptors including the calrecticulin/CD-91 complex and SPR-210 which are expressed on alveolar epithelial cells and macrophages, reviewed in (Jakel et al., 2013). The increased capacity of trimeric rfhSP-A to neutralise RSV as compared with nhSP-A may, therefore, be due to its capacity to neutralise RSV whilst lacking the N-terminal domain with potential to interact with cellular receptors and bring the virus into close proximity with potential sites for infection. The interaction of SP-A with putative receptors and its potential impact on RSV infection, however, remains to be fully characterised.

Dimeric rfhSP-A produced through a similar manner to functional trimeric rfhSP-A also reduced RSV infection to some degree, although this was significantly less effective than trimeric rfhSP-A. Interestingly, a monomeric SP-A CRD plus neck fragment has previously been shown to function in binding to alveolar type II cells and inhibit phospholipid secretion, suggesting that the trimeric structure with three correctly folded CRDs may not be essential for some of the broad functionality of SP-A at least (Murata et al., 1993).

In this present study, a homotrimeric fragment composed of only SP-A1 was used. However, human SP-A has previously been shown to be a mixture of SP-A1 and SP-A2 (Tagaram et al., 2007). Importantly, functional differences between SP-A1 and SP-A2 have been found, including the capacity of SP-A2 to bind various sugars with a higher affinity than SP-A1 (Sanchez-Barbero et al., 2007, Floros et al., 2009, Oberley and Snyder, 2003). Thus it would be interesting to produce a functional trimeric rfhSP-A from SP-A2 and compare its efficacy in neutralising RSV with the trimeric rfhSP-A of SP-A1 used in this study.

The importance of nhSP-A during RSV infection in an *in vivo* setting is likely different to *in vitro* due to the presence of lipid surfactant, immune cells including macrophages and T cells, other defence molecules and cytokines. nhSP-A likely has an important role in agglutination of RSV and, as previously suggested, may have a role in clearance by macrophages (LeVine et al., 1999). Further work comparing the capacity of trimeric rfhSP-A to prevent infection, enhance clearance by macrophages and reduce inflammatory pathogenesis in murine models of RSV infection is now needed.

## 5 Conclusion

We have for the first time implemented a novel expression tag to generate and successfully express a trimeric recombinant fragment of human SP-A; this tag may have general utility for expression of other heterologous protiens. Moreover, we have shown this trimeric rfhSP-A, to be highly efficacious at neutralising a clinically relevant strain of RSV in an *in vitro* model of human bronchial epithelial cells.

## Funding

This work was supported by the Medical Research Council (MRC), UK and the Swedish Research Council.

## Conflict of interest

None.
