# Supplementary material for: Novel expression of a functional trimeric fragment of human SP-A with efficacy in neutralisation of RSV
Source: Immunobiology. 2017 Feb;222(2):111–8. doi: 10.1016/j.imbio.2016.10.015 (PMC5152705; doi:10.1016/j.imbio.2016.10.015)
Supplement: Supplementary file 1 [file mmc1.docx]

# Supplementary Figure 1

M G H H H H H H M S H T T P W T N P G L A E N F M N S F M Q G L S S M P G F T A S Q L D D M S T I A Q S M V Q S I Q S L A A Q G R T S P N K L Q A L N M A F A S S M A E I A A S E E G G G S L S T K T S S I A S A M S N A F L Q T T G V V N Q P F I N E I T Q L V S M F A Q A G M N D V S A G N S A L V P R **G S P G I P G E C G E K G E P G E R G P P G L P** A H L D E E L Q A T L H D F R H Q I L Q T R G A L S L Q G S I M T V G E K V F S S N G Q S I T F D A I Q E A C A R A G G R I A V P R N P E E N E A I A S F V K K Y N T Y A Y V G L T E G P S P G D F R Y S D G T P V N Y T N W Y R G E P A G R G K E Q C V E M Y T D G Q W N D R N C L Y S R L T I C E F

# Supplementary Figure 2

| **A** | 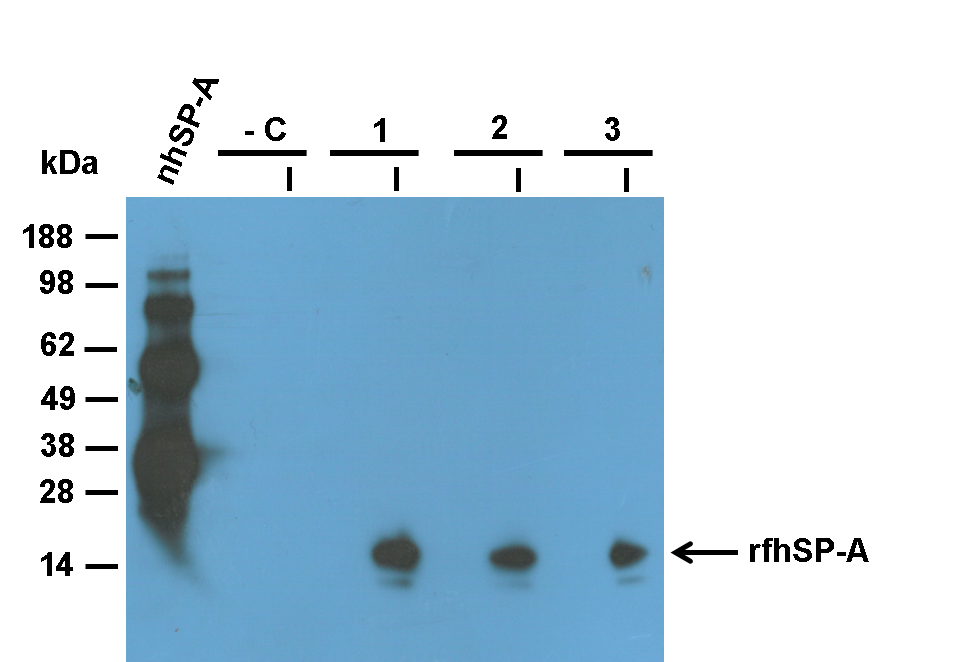 | **B** | 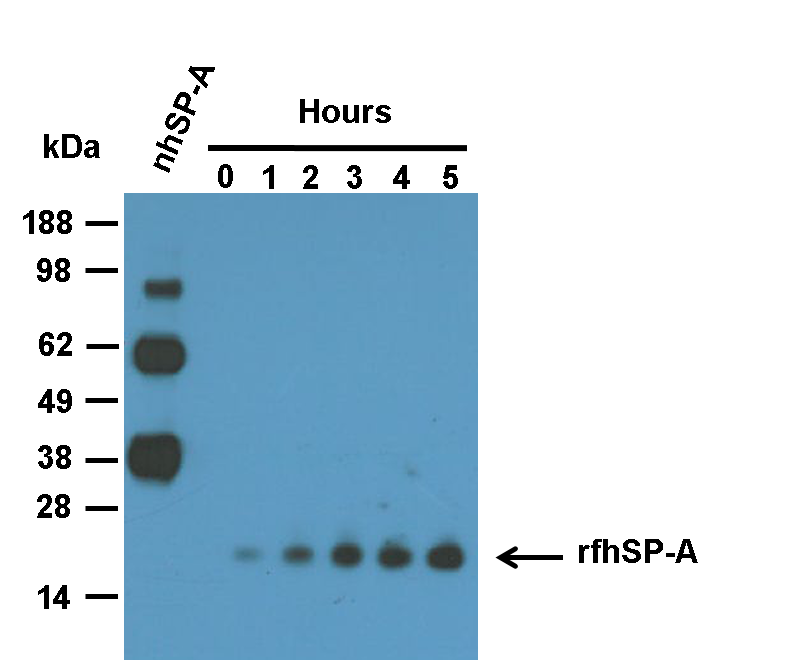 |
| --- | --- | --- | --- |
| **C** | 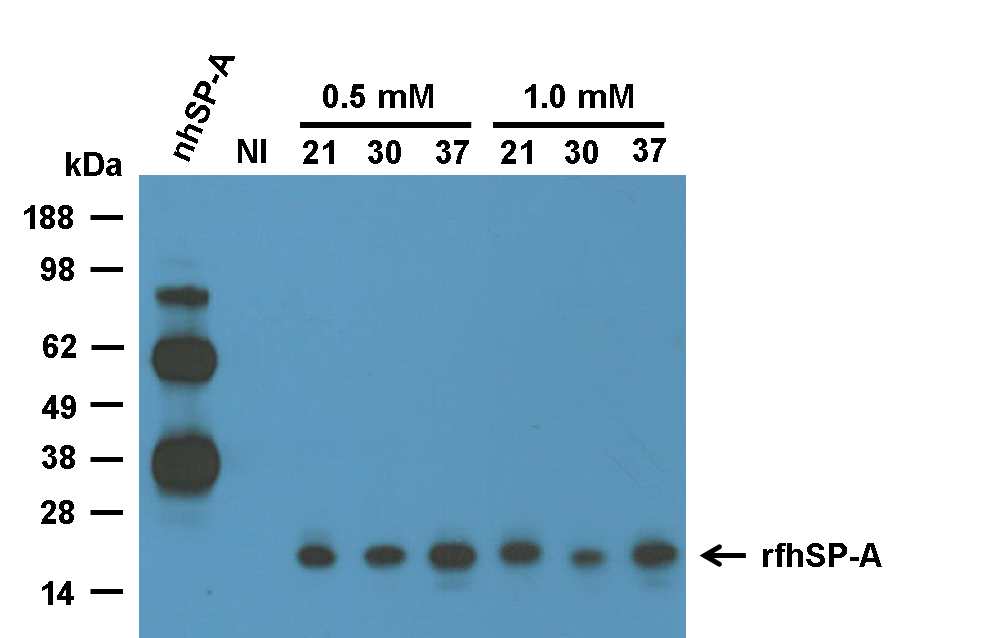 |  |  |

# Supplementary Figure 3

| **A** |  | **B** |  |
| --- | --- | --- | --- |
| **C** |  |  |  |





# Supplementary Figure 4

| **A** | 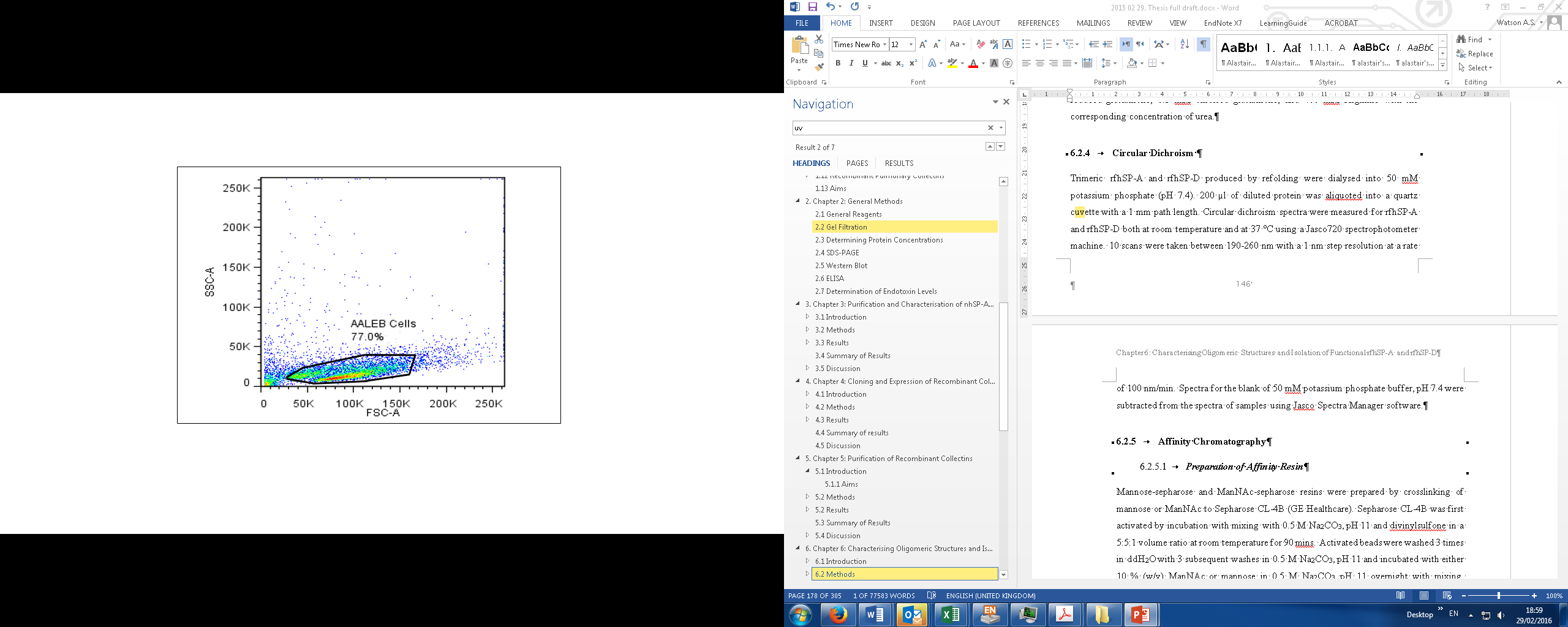 | **B** | **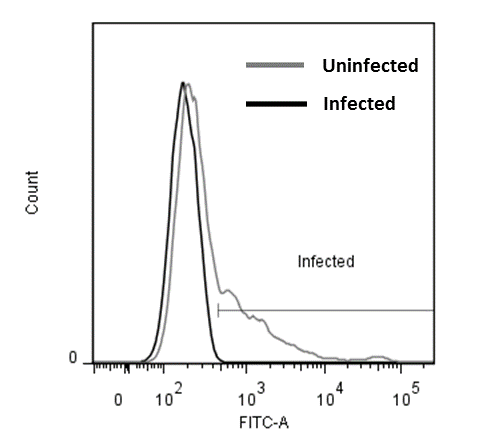** |
| --- | --- | --- | --- |
| **C** | **** | **D** | **** |
